# Wavelet-Based Analysis of Physical Activity and Sleep Movement Data from Wearable Sensors among Obese Adults

**DOI:** 10.3390/s19173710

**Published:** 2019-08-27

**Authors:** Rahul Soangra, Vennila Krishnan

**Affiliations:** 1 Department of Physical Therapy, Crean College of Health and Behavioral Sciences, Chapman University, Orange, CA 92618, USA; 2 Department of Physical Therapy, California State University, Long Beach, 1250 Bellflower Blvd, Long Beach, CA 90840, USA

**Keywords:** wearable sensors, obesity, wavelet analysis, longitudinal time series data

## Abstract

Decreased physical activity in obese individuals is associated with a prevalence of cardiovascular and metabolic disorders. Physicians usually recommend that obese individuals change their lifestyle, specifically changes in diet, exercise, and other physical activities for obesity management. Therefore, understanding physical activity and sleep behavior is an essential aspect of obesity management. With innovations in mobile and electronic health care technologies, wearable inertial sensors have been used extensively over the past decade for monitoring human activities. Despite significant progress with the wearable inertial sensing technology, there is a knowledge gap among researchers regarding how to analyze longitudinal multi-day inertial sensor data to explore activities of daily living (ADL) and sleep behavior. The purpose of this study was to explore new clinically relevant metrics using movement amplitude and frequency from longitudinal wearable sensor data in obese and non-obese young adults. We utilized wavelet analysis to determine movement frequencies on longitudinal multi-day wearable sensor data. In this study, we recruited 10 obese and 10 non-obese young subjects. We found that obese participants performed more low-frequency (0.1 Hz) movements and fewer movements of high frequency (1.1–1.4 Hz) compared to non-obese counterparts. Both obese and non-obese subjects were active during the 00:00–06:00 time interval. In addition, obesity affected sleep with significantly fewer transitions, and obese individuals showed low values of root mean square transition accelerations throughout the night. This study is critical for obesity management to prevent unhealthy weight gain by the recommendations of physical activity based on our results. Longitudinal multi-day monitoring using wearable sensors has great potential to be integrated into routine health care checkups to prevent obesity and promote physical activities.

## 1. Introduction

In the United States, about 66.3% of adults are overweight or obese [1]. The total medical expenditure associated with obesity exceeds $140 billion annually and represents approximately 9.1% of the total annual medical budget [2]. It is forecasted that by 2030, 51% of the total U.S. population will be obese, with a 33% increase in expected obesity prevalence and a 130% increase in severe obesity prevalence [3]. Considering gender-wise distribution, it is also estimated that the obesity rate in the United States will increase to approximately 50% for men and 52% for women, with the total number of obese people rising from 99 million in 2008 to 164 million by 2020 [4]. Obesity is a significant public health concern not only in the USA but also throughout the developed world [5]. Obesity is also a major risk factor for cardiovascular (CVD) diseases such as coronary heart disease (CHD), congested heart failure (CHF), stroke, ventricular dysfunction, and cardiac arrhythmias [6]. The degree of obesity is proportional to the risk of type 2 diabetes, CVD, certain types of known cancers, and even mortality [7,8]. Additionally, obesity is related to hypertension, with obese individuals being twice as likely to have high blood pressure compared to those of healthy weight [9], and obesity is considered the number one risk factor for stroke. 

Obesity is associated with adverse health consequences throughout the course of life [10] and severely affects the quality of life. It is found that having a body mass index (BMI) higher than 30 (obese) translates into a 200–300% higher mortality rate than that for healthy weight adults, and having a BMI of 25–29.9 (overweight) translates into a 20–40% higher mortality than that for normal-weight adults [11]. Physical activity (PA) is a modifiable risk factor for obesity [12]. However, the relationship between PA and obesity is not well understood. High PA levels should be an integral part of any treatment plan for obese individuals regardless of weight loss goals, and are associated with numerous CVD benefits [13]. Thus, monitoring PA and the development of new methods for longitudinal multiple day assessments of activities are essential for understanding the modifiable risk factors for obesity. Although PA is not the primary cause of the dramatic rise in obesity, promoting PA may be a broader solution to obesity prevention strategies. Some studies have reported that 70% of Americans do not get the recommended amount of PA to obtain any health benefits [14,15,16]. PA in human life is composed of different aspects such as transport-related, job or work-related, domestic work-related, and leisure time (playing). Currently, as per healthy activity and sleep guidelines, one is limited to 30 min/day of exercise and 7–8 h of sleep/day, leaving 16 h with no recommendation of sedentary behavior and its impact. Sitting time is an independent risk factor for the development of metabolic risk factors; thereby individuals with longer sitting times have worse metabolic profiles, even if they achieve the recommended amount of PA [17]. Some other studies utilized pedometers (step counter) and considered walking 5000 steps or less per day as an indicator of sedentary behavior [18], and 8000–10,000 steps as an indicator of a more active lifestyle. The role of light, moderate, and vigorous activity detection is vital to understand PA intensity in obese individuals. In addition to PA intensity [19,20] and sedentary behavior [21,22], there is a need to understand how these behaviors can be harnessed longitudinally throughout the day. Low-intensity daily activities (sedentary or sitting time) are related to cardiovascular disease and cancer [23]. Positive health outcomes directly relate to moderate and high-intensity activities [13], with a recommendation of 150 min/week. Besides, sleep is an essential factor associated with weight gain [24]. The general sleep recommendations are 7–8 h per night, and shorter or longer durations are associated with higher risk factors for a range of diseases [24,25,26,27].

Recently, the use of MEMS-based wearable inertial sensors has grown in both physical activity [28] and sleep research areas [29]. The objective assessment of activities and sleep behavior has traditionally been costly, difficult, or non-existent. Due to advancements in technology now, these measurements are possible using small wearable devices. The proliferation of wearable devices has led to the accurate, valid, and reliable measurement of movement activities. One of the important measurements of the inertial sensors is to detect periodic limb movements in sleep and sleep quality [30,31,32]. In addition, wearable sensors extensively monitor movement disorders [33,34]. Multi-day activity assessment was previously difficult to measure, and incorporating it into clinical research was limited because of unavailable measuring technology (limited memory and short battery life) and a lack of simple analytical methods to determine different activity levels and time spent in different activity levels in relation to health outcomes. Recent advancements in wearable technologies and the availability of sensors commercially has enabled longitudinally multi-day monitoring that allows concurrent assessments of sleep, stationary, and dynamic behaviors. However, there is a lack in the approach of these longitudinal multi-day inertial sensor data to explore activity and sleep behavioral monitoring. Importantly, the relations among obese individuals and their activity domains are not well understood. For example, PA behavioral information in obese individuals can be used as a treatment for poor sleep associated with obesity [35]. 

There is not much information about the magnitudes of PA and its patterns of PA on weight status. The purpose of this study is to compare the output from commercially available wearable devices using novel, simple data analysis methods for an objective assessment of activities and sleep behavior. In this study, we have determined new methods such as movement transition detection and wavelet analysis to measure the full day (multiple days) of activity performance behavior to guide future clinical reviews and recommendations. This study contributes to this debate by focusing on assessing PA objectively through obese and non-obese participants using wearable sensors.

## 2. Materials and Methods

The study was approved by Institutional Review Boards of Chapman University Participants with body mass index (BMI) measurements of 30 or above were classified as obese in the study. Twenty young adults consented to participate in this study (10 obese and 10 non-obese participants). The average age, height, and weight of the non-obese group was 24.7 ± 1.83 years, 166.8 ± 13.59 cm, and 67.1 ± 11.42 kg, and that for obese group was 25.6 ± 1.02 years, 163.4 ± 6.69 cm, and 89 ± 5.34 kg, respectively. Participants with any musculoskeletal or neuromuscular disorder were not included in this study. All the participants were healthy, young, and capable of following instructions. A body worn belt Move Monitor + Dynaport sensor (McRoberts, The Hague, The Netherlands; 85 × 58 ×11.5 mm, 74 g) collected data for 3-days. The device includes a triaxial accelerometer (sensor range and resolution: ±6 g and ±1 mg, respectively) and a triaxial gyroscope (sensor range and resolution: ±100 °/s and ±0.0069 °/s, respectively). The signals were recorded on an in-built SD (secure digital) memory card at a sample frequency of 100 Hz, and were later transferred to a personal computer for further analysis (using MATLAB, MathWorks, Natick, MA, USA). 

*Free-Living Activity Measurements*: Measuring the domains of activity behavior over the 24-h day (in multi-day assessment) are not limited to specific activities that can be measured in a laboratory, but is rather dependent on measuring free-living activities performed during daily living. In this study, we did not use any particular standards of activity for comparisons, nor was the analysis limited to laboratory-based gold standards, as in our previous studies [36]. All the sleep measurements were conducted using a single waist-worn wearable sensor, and analysis relied on an accelerometer-based measurement algorithm to estimate total sleep time. Sleep Analysis: Sleep overnight data was divided into four equal phases. We computed both the number of transitions during each phase of sleep and the resultant acceleration from the sensor:

(1) R_XYZ=√((〖AccX〗^2+〖AccY〗^2+〖AccZ〗^2))

**Figure 1:**
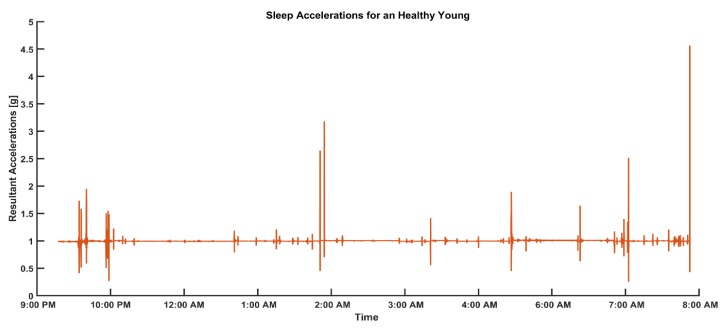
Representative data of resultant accelerations produced by sleep movements in healthy young adults.

Sleep data recognition from 3-days of acceleration data (Figure 1). We calculated the resultant acceleration in the XZ plane (denoted as R_A,xz_) of the sensor, which encounters accelerations due to gravity while sleeping. In contrast, the Z direction had the highest gravitational acceleration, and the X direction consisted of accelerations from the right and left directions of participants:

(2) $$R_{A,xz}=\sqrt{\left(A_{x}^{2}+A_{z}^{2}\right)}$$

A 1-s moving window evaluated the mean and variance of R_A,xz_ time series. If the window means of R_A,xz_ was bounded between 0.97 g and 1.02 g, the window variance was below 0.1 g, then, the data were categorized as sleep data (R_XYZ_Sleep). This was determined through a pilot study, where we found that breathing in subjects does not change their resultant acceleration more than 0.02 g at their lower back while laying down in supine, or on either side.

*Transition Peaks Evaluation:* Using sleep data (R_XYZ_Sleep), a moving window of 1 s will be used to evaluate all the maximas above the moving average by a threshold of 0.02 g. 

**Figure 2:**
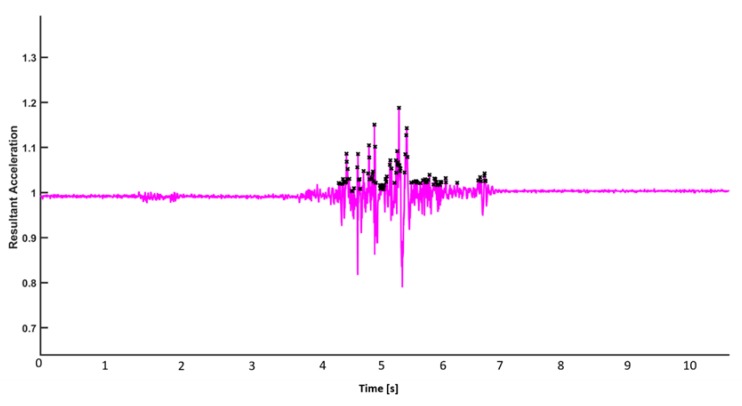
Representative figure for the identification of local maxima.

There are several local maxima (peaks) in one transition. The start and stop boundaries of these transitions are evaluated (Figure 2). 

**Figure 3:**
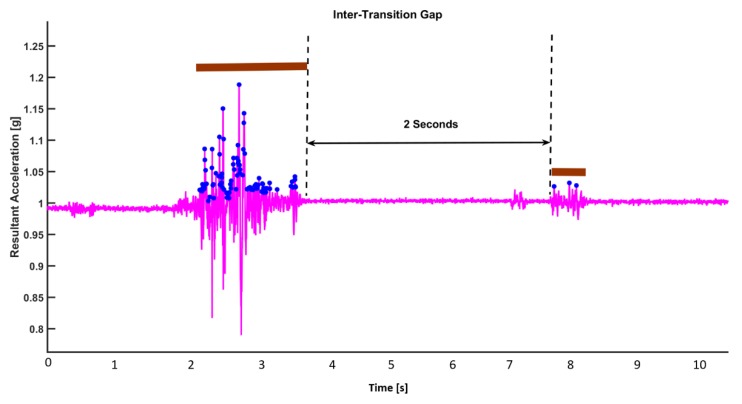
Inter-transition interval and transition durations in a transition.

*Categorization of Transition:* Each movement transition (bundle of local maxima) are differentiated using a simple algorithm. If the time gap between local peaks is less than 2 s, it is considered as being within the same transition. On the contrary, if the time gap between two consecutive local maxima is more than 2 s, the local maximas (peaks) are considered to be in separate transitions (Figure 3).

If two peaks are at least 2 s away in time from each other, we treat them as if they are in two different movements, and thus in different transitions. On the contrary, if two peaks are less than 2 s away in time from each other, we treat as if they are part of the same transition. With this declared definition, we firstly find all the transitions happening in the sleep dataset. Then, in each transition, we can gather information about the start and stop of each transition. 

**Figure 4:**
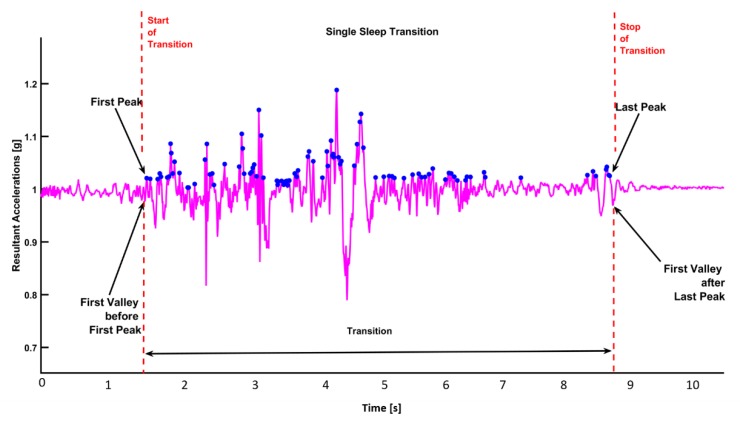
Start and stop events of a transition.

*Start and Stop of a Transition:* The starting point of a transition is defined as the first minima within an interval of 0.5 s before the first peak. Similarly, the stop point of a transition is defined as the first minima that occurs within 0.5 s after the last peak (Figure 4).

**Figure 5:**
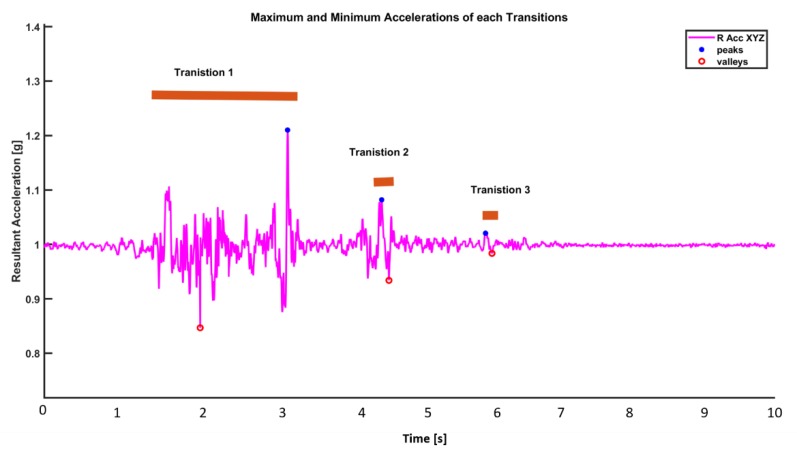
Maximum and minimum accelerations produced in a transition.

*Transition Duration:* It is defined as the time interval from the start of a transition to the stop of a transition. *Maximum and Minimum Acceleration Values in Transition:* The maximum acceleration is the maximum acceleration (maximum peak value) within the duration of a transition. The minimum acceleration is the minimum acceleration (minimum valley value) during a transition (Figure 5). Sleep parameters are defined in Table 1.

*Wavelet-Based Frequency Analysis:* For frequency analysis, Fourier transforms is the first choice to determine the frequency content of a signal. However, the frequency characteristics of inertial sensor signals change over time, and Fourier transform does not offer temporal localization. Wavelet transform is more promising in both the time and frequency domain information of the signal. *Creation of a Morlet Wavelet:* In order to make a Morlet wavelet, we can create a sine wave and a Gaussian wave and multiply them point by point. Both the sine wave and the Gaussian wave must have the same number of time points and the same sampling rate. The frequency of the wavelet is the frequency of the sine wave. The frequency of a Morlet wavelet (center frequency) is actually a band of frequencies.

The Gaussian window (bell-shaped or normal curve) is expressed as:

(3) $$Gaussian\;Window=ae^{-{\left(t\right)}^{2}/\left(2s^{2}\right)}$$

where the variable *a* is amplitude (height of the Gaussian curve), *t* is the time, and *s* is the standard deviation (width of Gaussian curve). The standard deviation s is defined as:

(4) $$s=\frac{n}{2\pi f}$$

where *f* is frequency (in Hertz), and *n* refers to the number of wavelet cycles. Wavelet cycles (n) define a trade-off between the temporal precision of a signal to its frequency precision. Although a real-valued Morlet wavelet is created by multiplying a sine wave by a Gaussian; for wavelet-based time-frequency analysis, we utilize a complex Morlet wavelet (cmw), which is created by multiplying a complex sine wave with a Gaussian wave.

Wavelet-based signal analysis in the time and frequency domain: In wavelet-based signal processing, the sampling rate of a wavelet is set to be equal to the sampling rate of the inertial sensor signals (f = 100 Hz). The wavelet time was chosen as 4 s, and the cycles of the Gaussian window are set to be from 12 to 14, which is relatively large, since we are also interested in the frequency domain information. The wavelet function can be expressed as:

(5) $$wavelet=e^{i2\pi f*t}*e^{-\;\frac{t^{2}}{2*{\left(\frac{cycle}{2\pi f}\right)}^{2}}}$$

**Figure 6:**
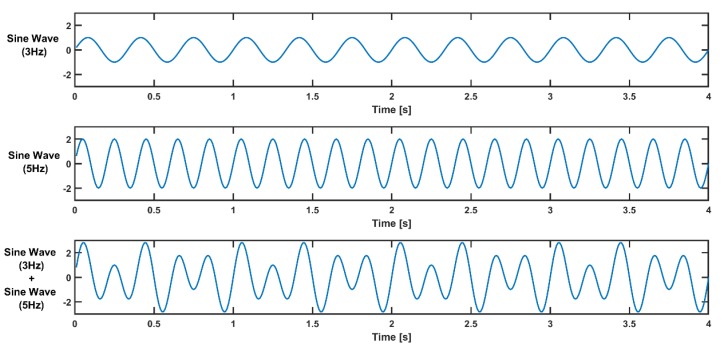
Simulated signal with two frequencies (3 Hz and 5 Hz) generated from a sine wave at 3 Hz and a sine wave at 5 Hz.

For example, to illustrate the wavelet-based time-frequency analysis method, we can simulate a signal containing only two frequencies (3 Hz and 5 Hz) for wavelet-based time-frequency analysis (Figure 6):

(6) $$\sin\left(2\pi f_{1}t\right)+2\sin\left(2\pi f_{2}t\right),\;0\leq t\leq 20,$$

where $f_{1}=3,\;f_{2}=5$.

The amplitude of a 3-Hz sine wave is 1, and that of a 5-Hz sine wave is 2.

**Figure 7:**
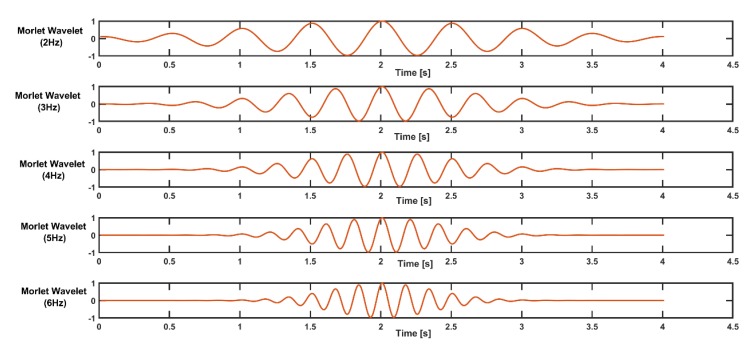
Morlet wavelets for different frequencies from 2 to 6 Hz.

Thus, a simulated signal wave carries higher power at a frequency of 5 Hz. In order to extract the frequency content (3 and 5 Hz) from the signal, a frequency band from 2 Hz to 6 Hz can be chosen for wavelet construction, with a step size of 1. The complex Morlet wavelets constructed are shown in the figure below (Figure 7).

**Figure 8:**
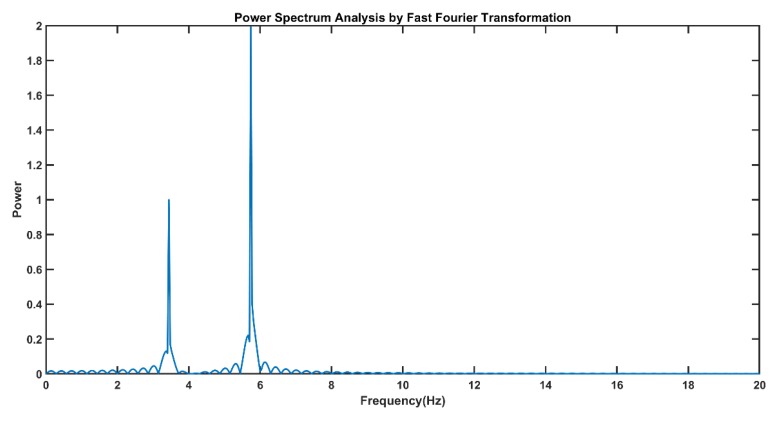
The fast Fourier transform (FFT)-based power spectrum shows that the simulated signal contains two frequencies at 3 Hz and 5 Hz, in which the power of the 5-Hz component is higher than that of the 3-Hz signal component.

The power spectrum from the fast Fourier transform (FFT) of the simulated signal is shown in the figure below (Figure 8). Power spectrum analysis using FFT does not provide temporal localization information about this simulated signal.

**Figure 9:**
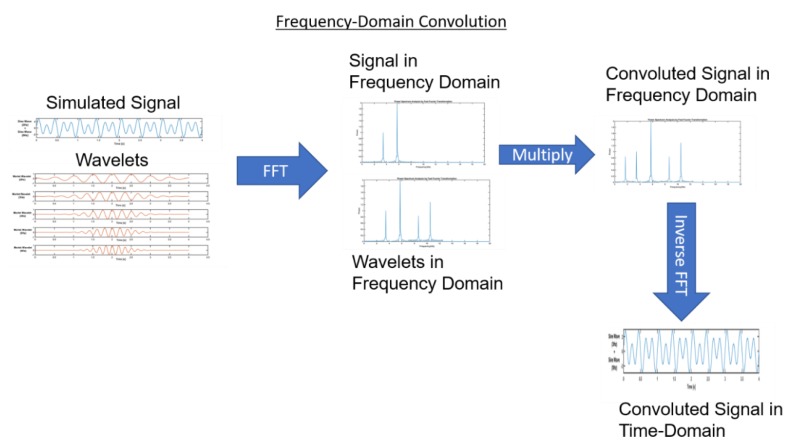
Illustration of the convolution theorem and frequency-domain multiplication.

Wavelet-based time-frequency analysis can be conducted by time-domain convolution between a signal and wavelets. Here, we performed frequency-domain convolution, since it is faster for long data sets. In order to perform a frequency domain convolution, firstly, FFT was performed on a signal and wavelets. This was followed by frequency-domain multiplication of the signal and wavelet FFT. This convoluted signal in the frequency domain provides complete information about the frequency at various temporal localizations. Inverse FFT can be performed to get a convoluted signal in the time domain (Figure 9).

**Figure 10:**
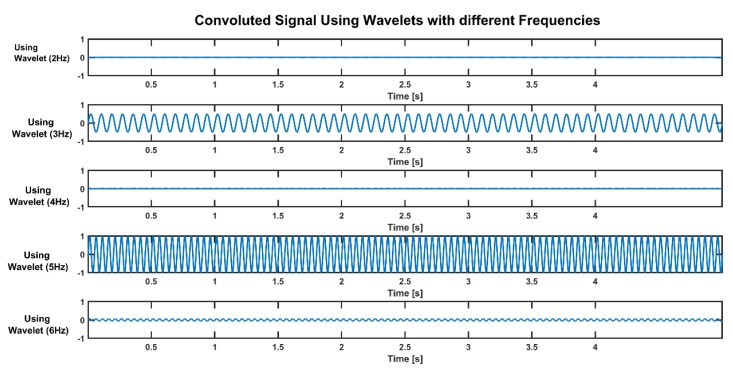
Convoluted signals using wavelets with different frequencies.

**Figure 11:**
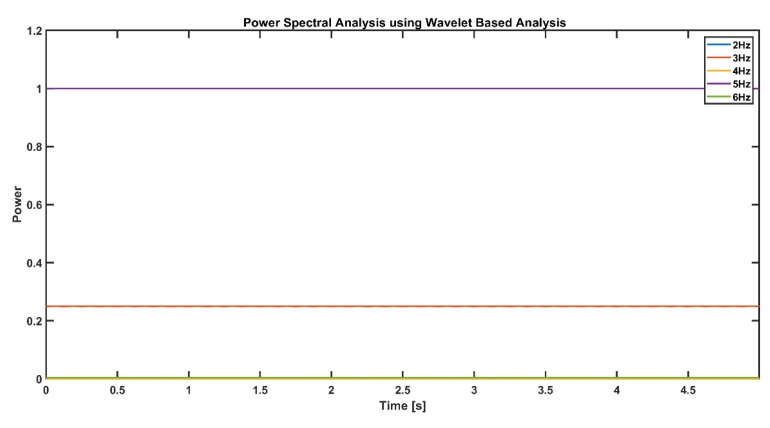
Frequency power of the 5-Hz signal component is 1, and that at 3 Hz is 0.25 throughout the time in the simulated signal.

As seen in figure above (Figure 10), only the 3-Hz and 5-Hz wavelet transforms show a convoluted sinusoid wave, and the amplitude of the 5-Hz signal is higher than the amplitude of the 3-Hz signal. Power spectral analysis of a convoluted signal is shown in the figure below (Figure 11). The frequency power of the 5-Hz signal component is 1, and for the 3-Hz component, it is 0.25. The power of a signal at a particular frequency is a function of its amplitude:

(7) $$power=\frac{amplitude^{2}}{2}$$

The Morlet wavelet transform-based convolution method is capable of analyzing the acceleration and angular velocity signals for longitudinal data. For a wavelet-based time-frequency analysis of longitudinal inertial sensor data, we have sub-categorized movements into three frequency bands where most of the daily activity movements occur. Each frequency band is subdivided into a total of five frequencies.

Band1: [0.1 0.2 0.3 0.4 0.5] Hz

Band2: [0.8 1.1 1.4 1.7 2] Hz 

Band3: [4 6 8 10 12] Hz

Wavelets within the band frequencies were convoluted with the inertial sensor signals to extract an accurate time and the related frequency of movement throughout the day. 

*Activities of Daily Living:* We computed Detrended Resultant Acceleration (DRA) signals for an analysis of magnitudes of activities of daily living. The resultant acceleration (RA,xyz) was defined as the resultant acceleration from all three unidirectional accelerometers.

(8) $$R_{A,xyz}=\sqrt{\left(A_{x}^{2}+A_{y}^{2}+A_{z}^{2}\right)}$$

(9) $$Detrended\;Resultant\;Acceleration\;\left(DRA\right)=\;\left|R_{A,xyz}-g\right|$$

where *g* is acceleration due to gravity. The one-second moving window size was chosen since activities such as sit-to-stand take about 2.2 s on average [37]. Through pilot testing, we found out that breathing and slight postural movements/adjustments such as head rotation and slight trunk movements produce mean DRA less than 20% of g (gravity) and standard deviations of less than 2% (<0.02 g). We also collected pilot data for walking trial signals from healthy young and older subjects, and found that normal walking DRA signals were below 50% of g and a standard deviation of less than 20% of g. Thus, we have categorized movement signals into three categories: low, medium, and high-amplitude movements (Table 2). Activity amplitude was defined as the time integral of DRA signals over the time span.

(10) $$Total\;Activity\;Amplitude=\int_{0}^{t}DRA$$

The total activity amplitude is defined as the integration of detrended resultant acceleration over the entire time of the day. The total activity amplitudes during the day were categorized into four time zone categories: Time zone 1 (TZ1) from 00:00 to 06:00, Time zone 2 (TZ2) from 06:00 to 12:00, Time zone 3 (TZ3) from 12:00 to 18:00, and Time zone 4 (TZ4) from 18:00 to 00:00. 

Statistical analysis was performed using JMP Software (JMP Pro, SAS Institute Inc., Cary, NC, USA, 2019). Descriptive analysis was performed for the total activity amplitude, power at different frequencies (0.1 Hz, 1.1 Hz, 1.4 Hz), number of transitions, root mean square (RMS) of transitions during sleep, number of hours of laying down, and number of transitions while lying down. For the analysis of these parameters, an approach of using Multiple Analysis of Variance (MANOVA) followed by univariate analysis was studied. Univariate ANOVA models was used to compare the endpoints among the two study groups (obese and non-obese) by an F test and between two groups by post hoc t-tests. Bonferroni corrections were applied for MANOVA results using JMP. 

## 3. Results

**Figure 12:**
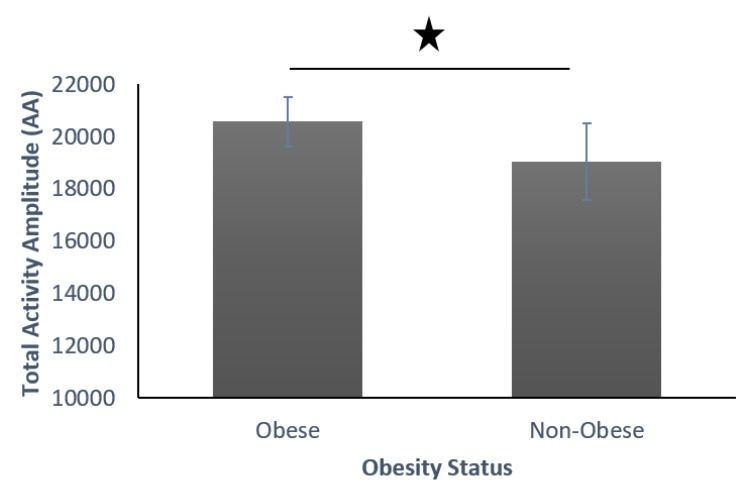
Total activity amplitude (AA) with low, medium, and high amplitudes among obese and non-obese participants.

**Figure 13:**
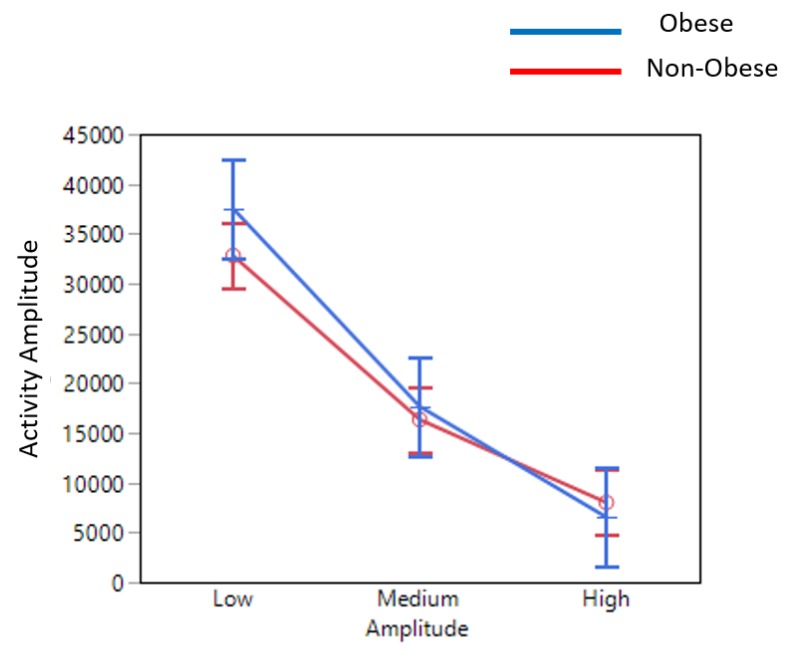
Activity amplitude (AA) of low, medium, and high amplitudes in obese and non-obese participants.

*Activities of Daily Living:* The results showed obese individuals producing significantly more transitions than their non-obese counterparts (Figure 12). However, upon looking into amplitude-wise movement status, obese individuals produced significantly more low-amplitude movements and significantly less high-amplitude movements (Figure 13). 

**Figure 14:**
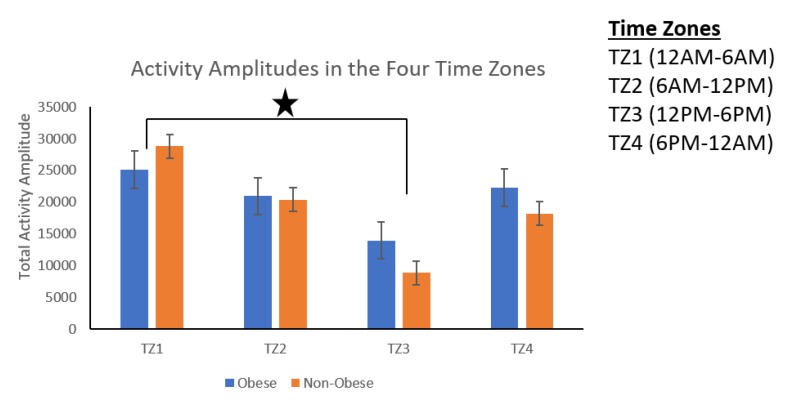
Time zone wise distribution of activity amplitude among obese and non-obese participants.

**Figure 15:**
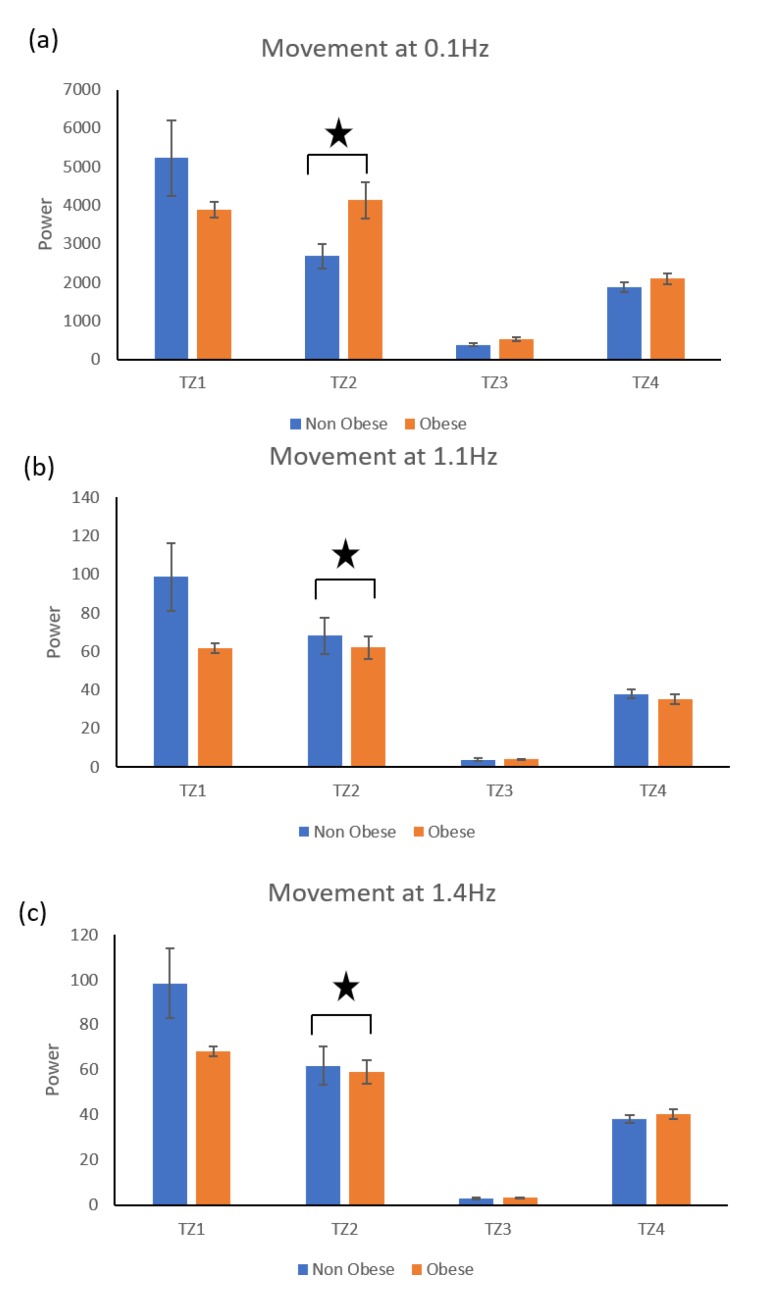
Amplitude of movement at low (0.1 Hz) and high (1.1 Hz and 1.4 Hz) frequencies.

The time zone wise distribution of activity amplitude (AA) shown in Figure 14 showed that the activity amplitude found in Time zone 1 (00:00 to 06:00) was significantly higher than the activity amplitude in Time zone 3 (12:00 to 18:00) for both obese and non-obese groups. *Movement Frequency Analysis*: In addition, obese group produced significantly more low-frequency movements, and the non-obese group produced significantly more high-frequency movements (Figure 15). 

**Figure 16:**
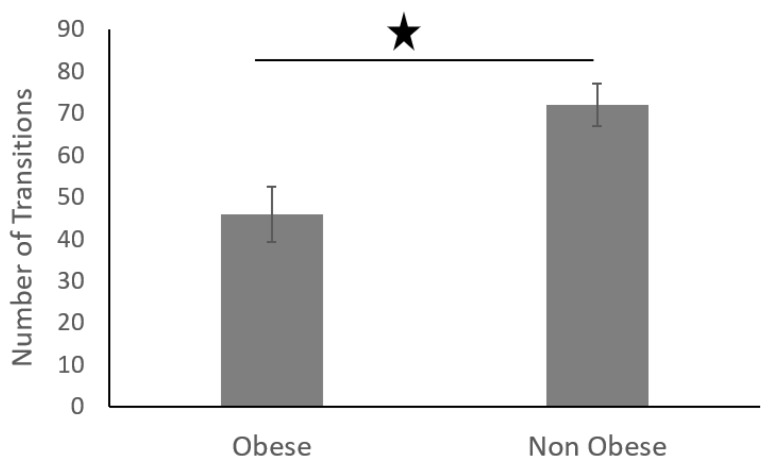
Average number of transitions in each phase of sleep.

*Sleep Data Analysis:* The average number of transitions in each phase during sleep in obese adults was significantly less than that of non-obese adults (Figure 16).

**Figure 17:**
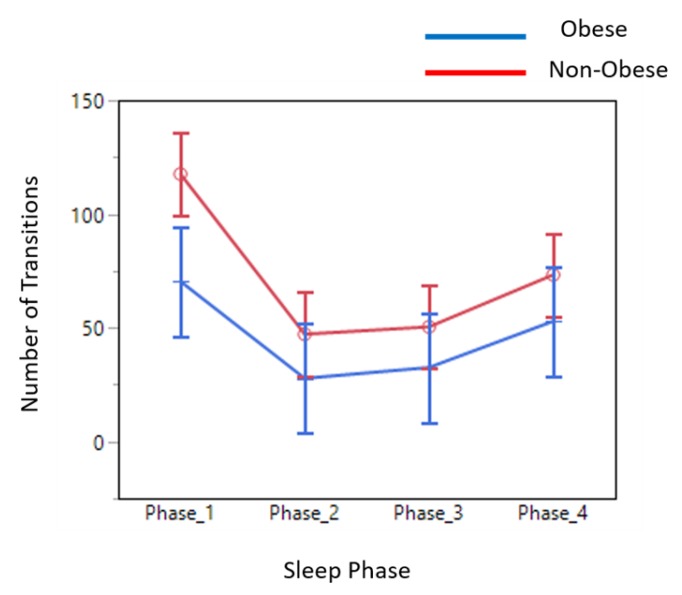
Number of transitions in each phase of sleep.

**Figure 18:**
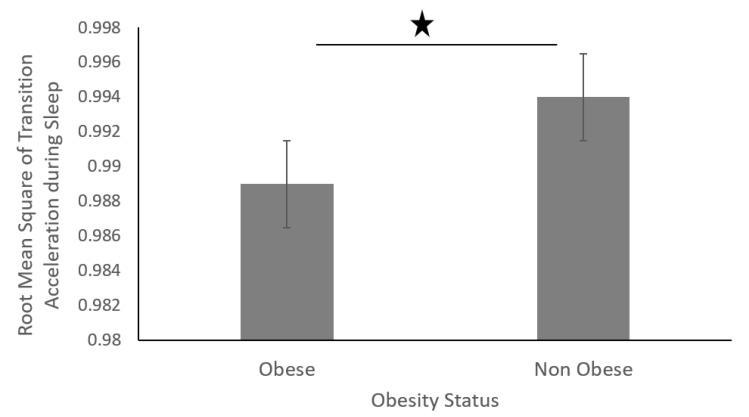
Root mean square of transition acceleration during sleep of obese and non-obese participants.

The sleep movement data was divided into four equal phases (phases 1–4) (Figure 17). The data showed that non-obese individuals produced a higher number of transitions across all four phases during their sleep. In addition, the root mean square of accelerations produced during the sleep transitions was significantly higher for non-obese individuals than that of obese individuals, as shown in figure below (Figure 18).

**Figure 19:**
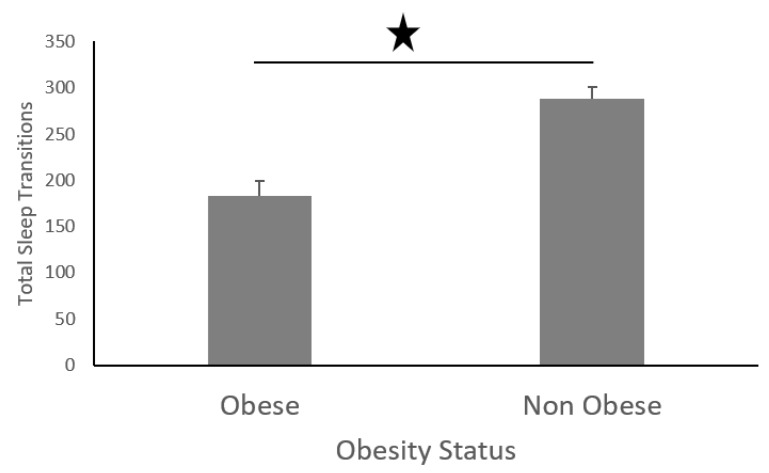
Total number of transitions in obese and non-obese individuals at night.

**Figure 20:**
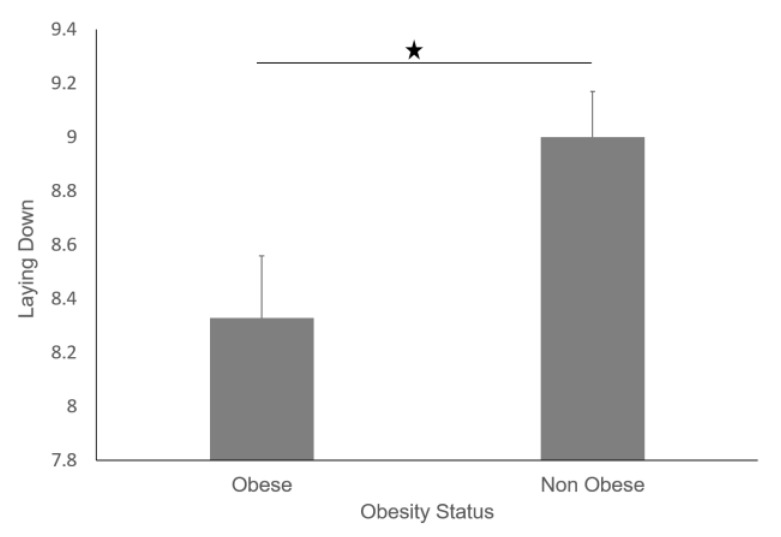
Average hours of laying down for obese and non-obese participants.

Furthermore, non-obese participants performed more sleep transitions than their obese counterparts (Figure 19), and non-obese participants also slept for longer (Figure 20).

## 4. Discussion

Obesity-related health care costs continue to rise for companies, totaling $15.4 billion in 2002 [38]. One of the biggest challenges for our society in the near future is to help the fast-growing obesity population to live independently in good health and with a good quality of life. New technologically-assisted approaches for obesity management have the potential to reduce this high cost. However, today, there is a knowledge gap between wearable technology-based longitudinal tracking and daily clinical use of the technology. In this study, we have explored how wearable inertial sensors can longitudinally track obese participants. To our knowledge, this is the first study investigating longitudinal three-day inertial data, and has developed new metrics dependent on the time and frequency domains, which could provide clinically relevant information for the management of obesity. The components of the multi-day longitudinal assessment are organized into domains of activity intensity (light, moderate, and high) and sleep behavior. 

We found that obese participants produced higher activity amplitude, but most of their activities were of low amplitude (Figure 12 and Figure 13). Many research studies have previously reported that obese people are found to have more strength [39,40], and are capable of producing higher activity amplitudes. We also found that the activity amplitudes were significantly higher in TZ1 (00:00–06:00) than in TZ3 (12:00–18:00) among both obese and non-obese groups. Recently, an article reported that women who are less active during morning hours may be at a higher risk of obesity [41]. In terms of frequency of movement, it was found that obese participants produced significantly more low-frequency (0.1 Hz) movements (Figure 15a), whereas non-obese counterparts produced significantly more high-frequency (1.1 Hz and 1.4 Hz) movements (Figure 15b,c). This is congruent with other studies that have reported that obesity has deleterious effects on motor control, thereby affecting the efficient execution of daily living activities [42]. From the sleep data analysis, we show that non-obese participants made a significantly higher number of transitions during all four phases of sleep (Figure 16 and Figure 17). We also showed that the root mean square of transition acceleration during sleep among non-obese participants was significantly higher than that of obese participants, thus affecting the quality of sleep. We also found that the total number of transitions during the night were significantly less for obese individuals (Figure 19) and their average sleep time was less than that of the non-obese participants (8.3 h versus 9 h) (Figure 20). An association between sleep and obesity is well known [43,44]. On the onset of obesity, regular PA remains extremely low [45]. Our findings provide convincing evidence that obese individuals exhibited low-frequency movements, which may lead to a self-perpetuating vicious circle of fewer activities of daily living with low overall energy expenditure and increasing weight gain and obesity. This study establishes a baseline for longitudinal assessments among obese and non-obese individuals. Such baselines are important for designing interventions for obesity management using wearable sensors.

## 5. Limitations

All previous studies have looked into activity detection and energy expenditure utilizing wearable sensors. In this study, we have adopted a new approach to utilizing time and frequency-based signal information for longitudinal data. There is a preponderance of scientific literature supporting PA as an important lifestyle behavior for the management of obesity. Thus, it is crucial to adopt wearable technology that may enhance engagement in PA in overweight or obese individuals. The study is limited, since the data cannot be compared with existing literature. Finally, this study was conducted in a small, young, healthy sample of 20 people (10 obese and 10 non-obese participants), and therefore, findings are not generalizable to other populations. In addition, there are several confounding factors between PA and increased body weight. A few of them include age, education, smoking, and ethnicity. 

## 6. Conclusions

Mobile health utilizing wearable technology is a growing endeavor to improve healthcare services. This study reports the feasibility of wearable sensor-based longitudinal tracking, which can be helpful to provide a quantitative assessment of activities of daily living and sleep behavior among obese and non-obese groups. We found that obese individuals produced more movements of low frequency compared to non-obese individuals, who produce high-frequency movements. The longitudinal tracking using wearable inertial sensors enables for continuous long-term activity monitoring in the free-living home environments of obese and non-obese groups. This is one of the first studies looking into the frequency-domain and time-domain movement features of ADLs. Such assessments can be widely adopted as an assistive tool for obesity management. A global aim is to use this technology to reduce health disparities, especially among obese patients, and lower the long-term cost of more personalized care. These findings also provide valuable information for the development of new wearable sensors, which can be utilized for obesity management. More research is necessary for studying obesity in other populations and for studying comparisons across different study designs.

## Figures and Tables

**Table 1:** Parameters and their definitions.

Parameter	Definition
**Number_of_Transitions**	The total number of transitions found in one sleep dataset.
**Sleep_Hours**	Total number of hours of sleep
**Transition_Max_Acc**	Maximum of acceleration value in each transition
**Transition_Min_Acc**	Minimum of acceleration value in each transition
**Transition_RMS**	Root mean square of acceleration during a transition
**Transition_Range**	Difference between max and min acceleration value in each transition
**Transition_Duration**	Average time for each transition
**Total Activity Amplitude**	

**Table 2:** Categorization of Detrended Resultant Acceleration (DRA) signals into low, medium, and high-amplitude signals.

Low		Medium		High	
Mean	Standard Deviation	Mean	Standard Deviation	Mean	Standard Deviation
m_DRA <0.2 g	std_DRA <0.02 g	0.2 g < m_DRA < 0.5 g	0.02 g < std_DRA < 0.2 g	m_DRA >0.5 g	std_DRA >0.2 g
